# Forty‐eight‐year‐old female MUTYH carrier presenting with five concurrent primary cancers

**DOI:** 10.1002/cnr2.1455

**Published:** 2021-06-26

**Authors:** Aaron Arroyave, Laurentia Nodit, Devin Clegg, Andrew Russ

**Affiliations:** ^1^ Department of Surgery University of Tennessee Medical Center Knoxville Tennessee USA; ^2^ Department of Pathology University of Tennessee Medical Center Knoxville Tennessee USA; ^3^ Department of Surgery, Colon and Rectal Surgery University of Tennessee Medical Center Knoxville Tennessee USA

**Keywords:** colon cancer, genetics, gynecology, oncology

## Abstract

**Background:**

MUTYH‐associated polyposis is a rare disorder resulting from mutations involved in DNA mismatch repair. This results in an increased susceptibility to colonic adenomatosis and other cancers. Studies have examined the resulting frequency of extracolonic manifestations; however, these typically occur alone, concurrently, or temporally separate from an already diagnosed colorectal cancer in individuals with a biallelic mutation.

**Case:**

Reported here is a case of five distinct primary neoplasms presenting simultaneously in a patient monoallelic for an MYH mutation. These neoplasms included squamous cell carcinoma of the vulva, rectal adenocarcinoma, synchronous anal adenocarcinoma, papillary thyroid carcinoma, and ovarian serous psammocarcinoma. Throughout her course, she underwent multiple surgical procedures, neoadjuvant chemoradiation, with further adjuvant therapy, and treatment ongoing. Due to her unique presentation, she underwent genetic testing that demonstrated she was monoallelic for an MYH mutation.

**Conclusion:**

The patient had a positive response to her treatment and surgical procedures with ongoing adjuvant therapy. She will continue to undergo further genetic testing, and testing for her children is being considered. This case demonstrates a unique presentation associated with a monoallelic MYH mutation that is not described in the current literature and warrants further investigation.

## 1 INTRODUCTION

First described in 2002, MUTYH‐associated polyposis (MAP) is an autosomal recessive familial colorectal cancer syndrome due to a mutation in the MYH gene.^1^, ^2^ Through its excision of misincorporated nucleotide residues caused by dihydro2′deoxyguanosine, a mutagenic product of oxidative DNA damage, adenine glycosylase, the enzyme encoded by this gene, functions in DNA mismatch repair.^1^, ^3^, ^4^, ^5^, ^6^ When pathogenic MYH mutations occur, the phenotype expressed is typically characterized by extensive colonic adenomatosis and an increased risk of colorectal and extracolonic malignancies. Colorectal cancer risk is increased between 23‐ and 28‐fold in biallelic individuals. The presence of an increased risk of malignancy associated with mono‐allelic mutations, particularly of the G396D variant seen in our patient, remains controversial.^7^ The majority of current studies surrounding this topic sought to delineate the characteristics of this disease by identifying MYH mutations in populations of patients with polyposis and without an identified familial syndrome.

Concurrent extracolonic malignancies were frequently encountered^2^; however, none as extensive as those was seen in the case reported herein. Additionally, extensive colonic adenomatosis has historically been pathognomonic for familial adenomatous polyposis. However, although only one gastrointestinal polyp was encountered in our case, studies have positively correlated the extent of polyposis with an increased likelihood of harboring an MYH mutation.^8^, ^9^, ^10^ Therefore, in patients with multiple primary cancers, and patients with polyposis without an associated APC mutation, further genetic testing should be strongly considered.

Due to the rarity of MAP, specific clinical screening and treatment guidelines have yet to be published. In light of this, the disparate and aggressive presentation of this case serves two educational purposes. These are to highlight the importance of a thorough workup in patients with oncologic presentations suspicious for genetic aberration, and how variable expressivity can lead to unpredictable presentations necessitating a broad differential diagnosis.

## 2 CASE PRESENTATION

**FIGURE 1:**
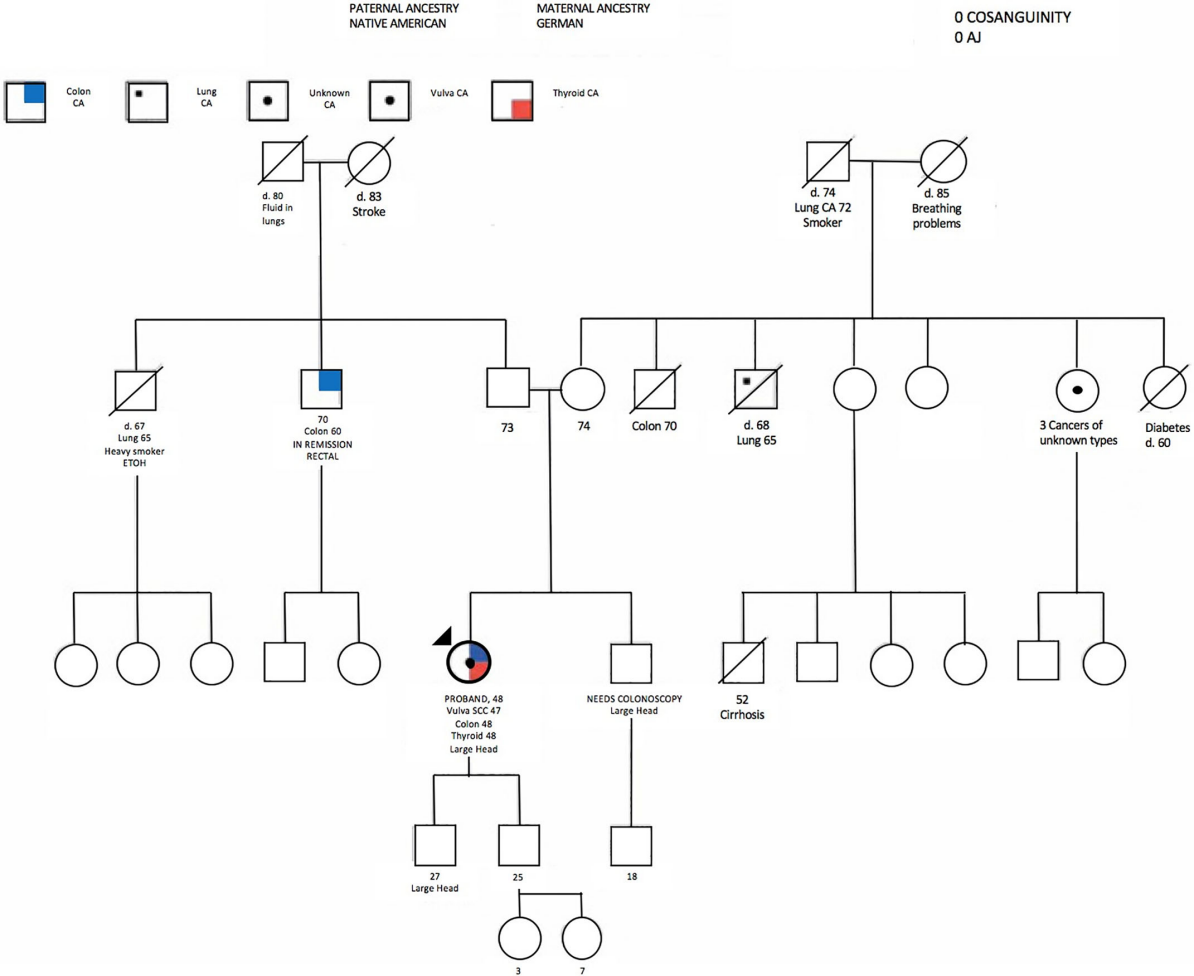
Pedigree

A 48‐year‐old female with family history of colon, rectal, and lung cancer (Figure 1), and a past medical history of obesity and type 2 diabetes mellitus, presented in July 2018 with a several‐month history of an enlarging pruritic labial mass. This was found to represent a high‐grade squamous intraepithelial neoplasm for which a radical right partial vulvectomy was performed. Final pathology demonstrated a stage IB squamous cell carcinoma. Due to the presence of bilateral inguinal lymph node enlargement, a bilateral inguinal lymph node dissection was performed which demonstrated that all 15 lymph nodes removed were negative. At follow‐up, the patient complained of fecal incontinence. To evaluate this, upper and lower endoscopies were performed which demonstrated a large rectal mass representing adenocarcinoma characterized by a low probability of microsatellite instability and an anal mass representing a second adenocarcinoma. No polyps were encountered.

**FIGURE 2:**
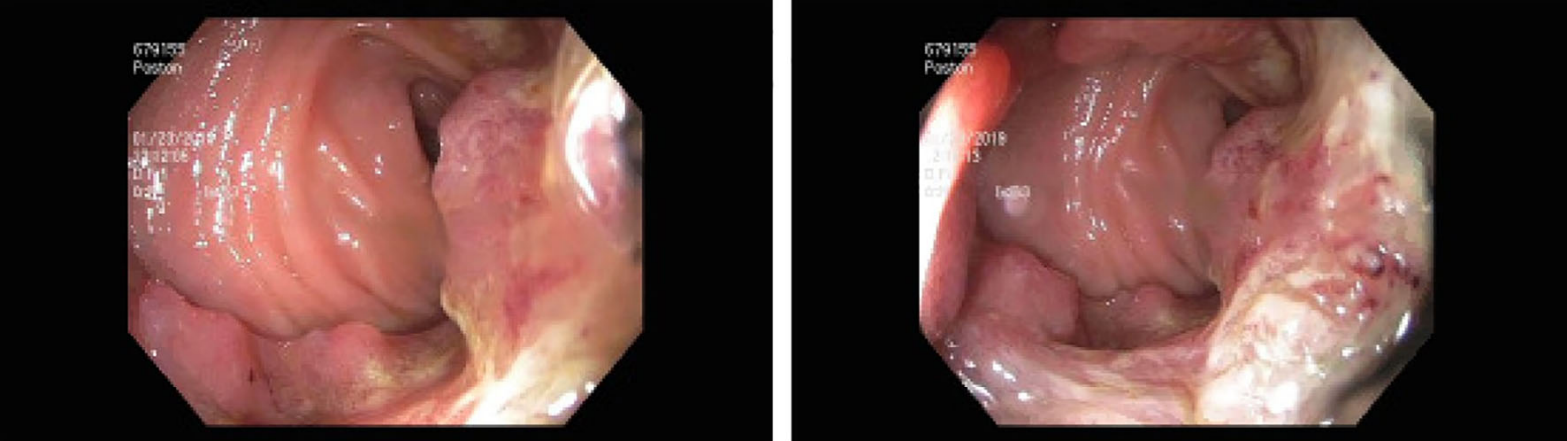
Anorectal mass on colonoscopy characterized as fungating, infiltrative, polypoid, sessile, ulcerative, nonobstructing, and circumferential

**FIGURE 3:**
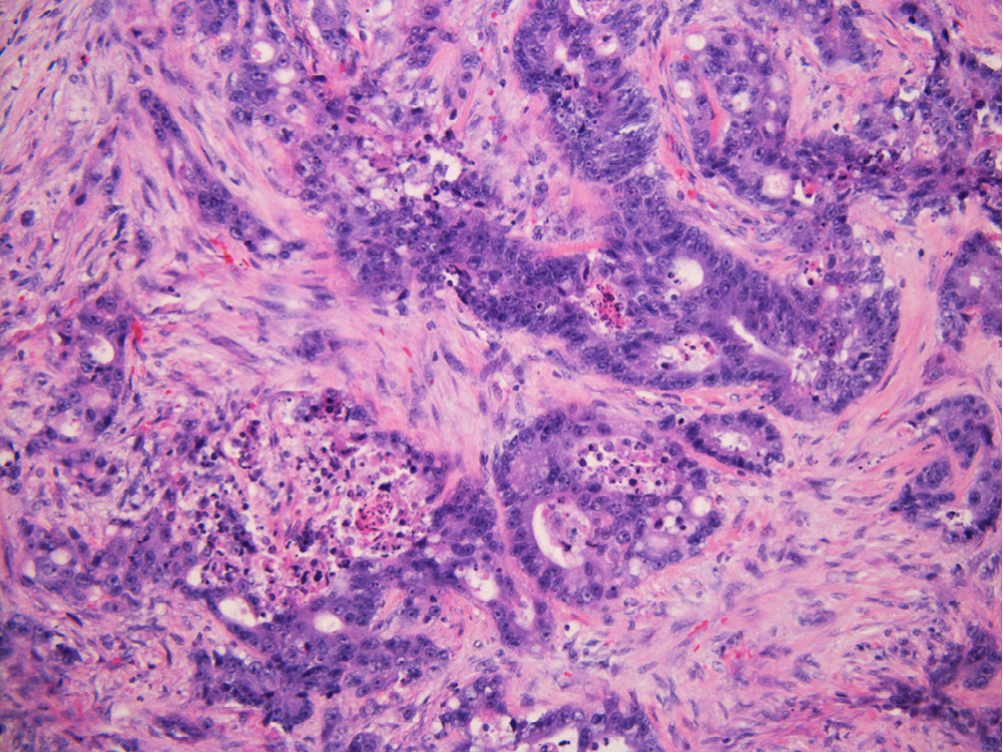
Microscopic photograph showing adenocarcinoma. Hematoxylin‐eosin stain, ×200 magnification

**FIGURE 4:**
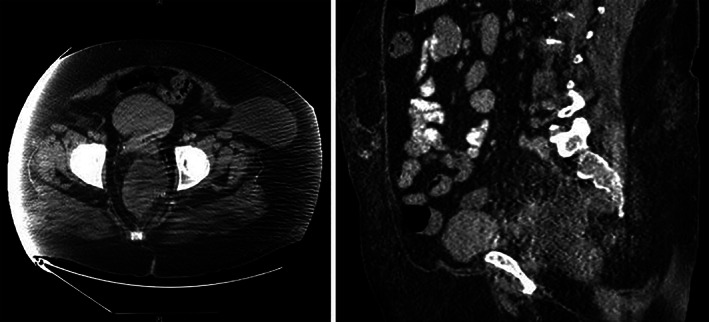
CT chest/abdomen/pelvis: Anorectal mass axial and sagittal view

**FIGURE 5:**
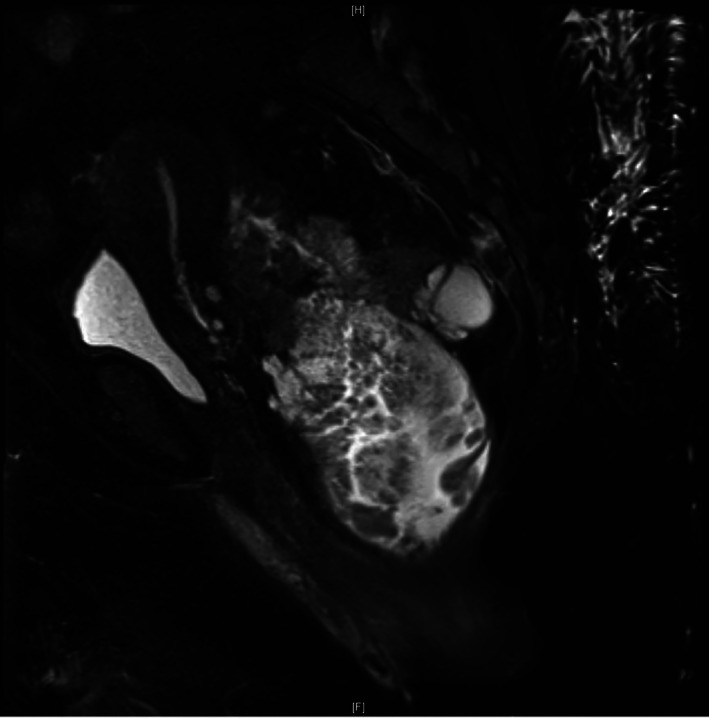
MRI pelvis demonstrated a circumferential, fungating, mucinous, 15.6 cm mass within 1 cm of the anal verge, extending from above the peritoneal reflection down to the lower rectum and upper anal canal. The tumor involved the puborectalis, and internal and external anal sphincters, and was noted to have a 50‐mm depth of invasion. Due to abutment of the posterior cervix, vaginal cuff, and bilateral levator muscles, the tumor was believed to invade adjacent organs and was staged as a T4b tumor. The MRI was also suspicious for local lymph node involvement; therefore, the patient was deemed to be clinical stage IIIC (T4b, N1, Mx)

**FIGURE 6:**
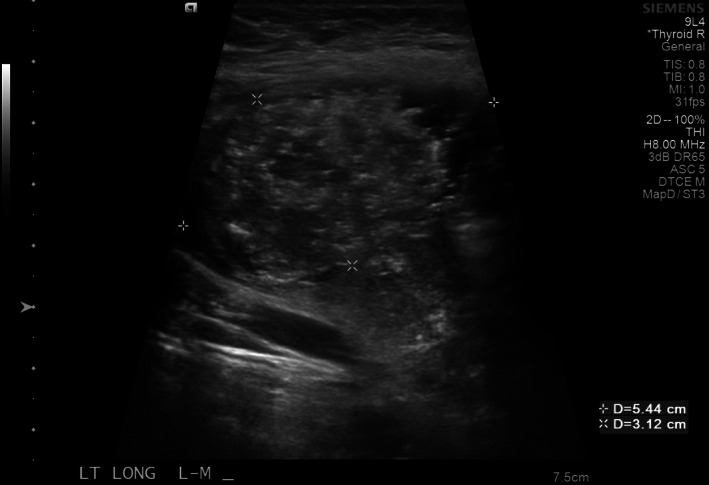
Thyroid ultrasound demonstrating multiple bilateral nodules concerning for malignancy. Pictured below is a TI‐RADS 5, left‐sided thyroid nodule, approximately 5.4 cm in greatest dimension

In January 2019, colonoscopy revealed a large, circumferential, fungating, infiltrative, ulcerative, and nonobstructing rectal mass (Figure 2). Biopsies of this mass demonstrated adenocarcinoma (Figure 3). In February 2019, further staging work‐up included an elevated CEA level of 416.9 ng/mL, a CT chest/abdomen/pelvis, and a pelvic MRI. In addition to demonstrating the rectal and anal neoplasms, (Figure 4), the CT chest/abdomen/pelvis demonstrated a multinodular thyroid, a 1.5 cm right adrenal adenoma, a thin‐walled pulmonary nodule or cyst, bilateral iliac chain lymph node enlargement, a single enlarged portocaval lymph node, and a large ventral hernia. Staging pelvic MRI redemonstrated the rectal lesion which was noted to be 15.6 cm in size, invading 50 mm in depth, extending from above the peritoneal reflection down to within 1 cm of the anal verge, possibly involving the pelvic floor musculature, posterior cervix, and vaginal cuff. Local lymph node involvement was radiographically suspected (Figure 5). Due to concern for local lymph node involvement and extracolonic extension, the patient was in clinical stage IIIC (T4b, N1, Mx). The asymptomatic thyroid mass was evaluated via ultrasonography which demonstrated two TI‐RADS 5 left‐sided thyroid nodules and a TI‐RADS 4 right‐sided thyroid nodule (Figure 6). Left lobe thyroid biopsies demonstrated papillary thyroid carcinoma.

Due to the presence of multiple primaries, genetic testing was performed and demonstrated heterozygosity for a p.G396D MUTYH mutation. The patient was negative for a multitude of other mutations including APC, MLH1, MSH2, MSH6, PMS2, BRCA 1&2, MEN1, RET, PTEN, and TP53. Counseling was provided regarding associated risks, and early screening colonoscopy for her offspring was recommended. The presentation of colorectal cancer alongside extracolonic malignancies raises suspicion for germline mutations and familial cancer syndromes.

**FIGURE 7:**
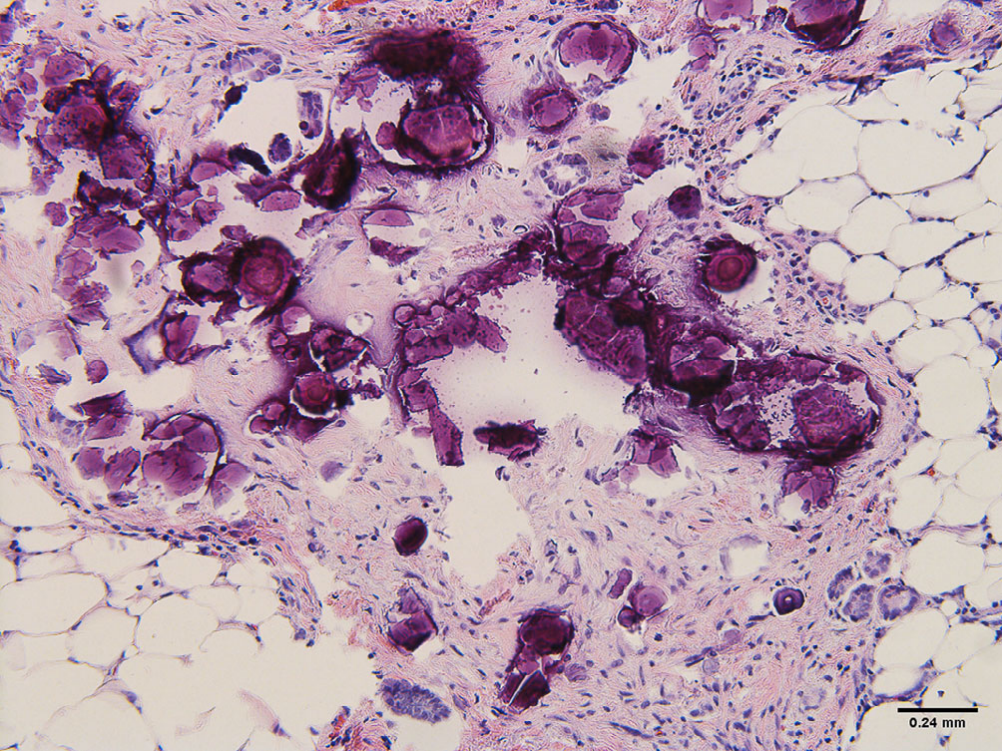
Serous peritoneal psammocarcinoma histology stained positive for MOC 31, Ber‐EP4, WT‐1, PAX8, and estrogen receptor. Stained negative for calretinin, D2‐40, p53, and TTF‐1

The patient underwent neoadjuvant chemoradiation prior to undergoing a total thyroidectomy with central lymph node dissection. Papillary carcinoma was found within both lobes, and three of nine lymph nodes were positive for associated metastases. Radioactive iodine ablation was planned. A follow‐up CT scan in May demonstrated a response by the rectal tumor to neoadjuvant chemoradiation, with a decrease in size of the rectal tumor from 8.7 × 7.7 × 10.7 cm to 6.6 × 5.0 × 6.9 cm (Figure 6). In June, the patient underwent a posterior pelvic exenteration with a total abdominal hysterectomy and bilateral salpingo‐oophorectomy, as well as a concurrent sigmoid colectomy with en bloc radical vaginectomy and perineal proctectomy. No gross metastatic foci were encountered intraoperatively, and the tumor was noted to be confined to the anorectum during resection. The wound was closed primarily, a pelvic drain was placed, and a ventral hernia repair was performed. A single hyperplastic polyp was found in the colon. In addition to the two separate adenocarcinomas in the anus and rectum, a left ovarian serous implant of psammocarcinoma (Figure 7) was identified. Scattered foci of low‐grade serous carcinoma (psammomatous carcinoma) were found within the ventral hernia sac tissue. All 22 lymph nodes were negative for malignancy. The patient was discharged on postoperative day 3 after an uncomplicated hospital stay.

**FIGURE 8:**
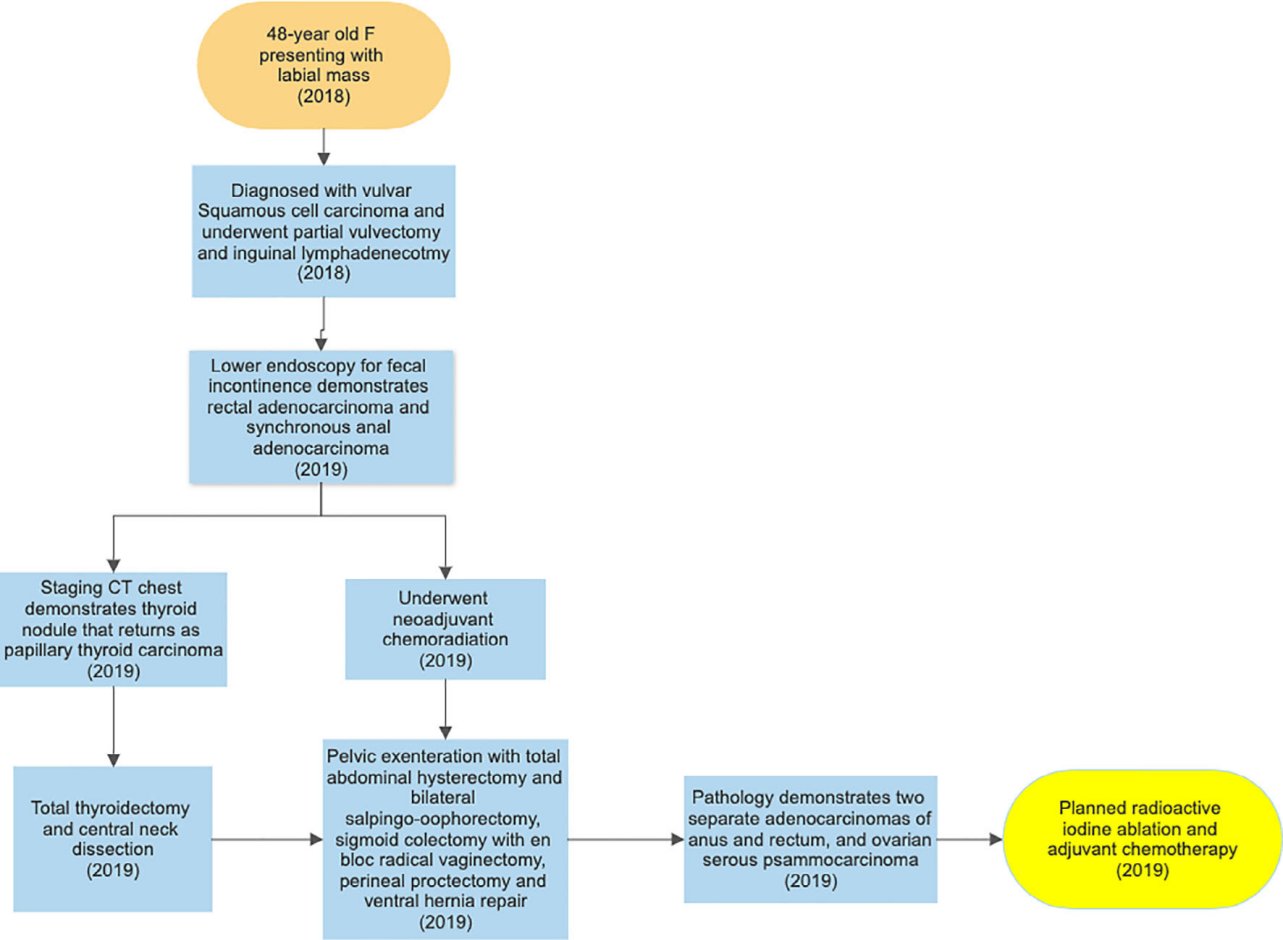
Summary of patient's clinical course and timeline of diagnoses

The patient was discharged to a skilled nursing facility for continued wound care. Ongoing plans include adjuvant therapy for her anorectal cancer, as well as further therapy to treat her low‐grade peritoneal primary serous cancer. This patient's clinical course and timeline of diagnoses are summarized in Figure 8. This patient's disease was investigated by both our genetics and dermatology departments thoroughly and was presented at a multidisciplinary conference to discuss the possibility of Cowden syndrome. They further recommended obtaining genetic testing for her children which is being considered.

## 3 DISCUSSION

The case presented contains several peculiarities which merit discussion and defy the currently tenuous conclusions surrounding this rare disease. The presentation of our patient raised concern for Lynch syndrome, Cowden disease, familial adenomatous polyposis (FAP), and MYH‐associated polyposis. The presentation of Lynch syndrome is characterized by the development of colorectal cancers in addition to other extraintestinal cancer, including ovarian cancer. This coupled with the relatively low number of intestinal polyps seen in Lynch syndrome, consistent with our patient, further raised suspicion for this diagnosis.

However, immunohistochemical stains for MLH1, MSH2, MSH6, and PMS2, which are used to screen for Lynch syndrome, were negative. Additionally, though the patient did not demonstrate any germline mutations associated with Cowden syndrome, the diagnosis was considered due to her medical history of colon cancer, papillary thyroid cancer, and thyroid structural disease. However, the patient did not meet diagnostic criteria based on clinical history alone. In the current literature, MAP is most commonly diagnosed within patients suspected of having FAP but without an APC mutation. While attenuated FAP (AFAP) is characterized by a low number of polyps, absence of an APC mutation, again, excluded this diagnosis. The finding of a p.G396D MUTYH mutation indicated the most likely diagnosis to be MAP. This disorder is genetically defined by a homozygous mutation, and therefore, our patient, by current standards, should only be carrier and should not phenotypically express this typically Mendelian autosomal recessive disorder.

The two most common MYH mutations, of the greater than 60 mutations currently identified, include Y179C and G396D mutations.^11^ In the current literature, the risk of colorectal cancer due to a biallelic MYH mutation is increased between 23‐ and 28‐fold, depending on the specific mutations. However, the presence of an increased risk of malignancy associated with monoallelic mutations, particularly of the less virulent G396D variant seen in our patient, has yet to be established.^7^ While G396D mutations, and the types of extracolonic malignancies which developed in our patient, are characteristic of MAP, the heterozygosity of her mutation might indicate an additional unidentified genetic aberration resulting in an increased susceptibility to malignant degeneration.

In some respects, the clinical presentation of our patient conforms strongly with the diagnosis of MAP, especially with regards to the types of extracolonic malignancies developed. Ovarian, thyroid, and skin cancers have all been found in individuals with MAP‐associated colorectal cancer.^2^, ^12^, ^13^, ^14^ The risks of developing extracolonic malignancies have not been respectively identified. However, Vogt et al. calculated a 38% overall lifetime risk of developing extracolonic malignancy in patients with MAP. The relative absence of polyps, as seen in our patient, is uncharacteristic of MAP. A study by Wang et al. found that the presence of a biallelic germline MYH mutation correlated with the presence of ≥20 adenomatous polyps.^9^ In one study of 152 patients with polyposis, among patients with adenomas ranging in number between 3 and 100, biallelic MYH mutations were found only in individuals with greater than 15 polyps.^10^ Additionally, of the 25 MAP patients within the study by Sampson et al., 11 patients had between 10 and 100 adenomas, nine had greater than 100, and five had adenomas too numerous to count.^8^ This unique presentation of an MUTYH mutation raises suspicion for concurrent and undetected genetic abnormalities. However, because MAP remains rare, and consequentially, studies regarding its prevalence and natural history remain relatively inconclusive, it is possible that this case only highlights how much left there is to learn about this disease. Formal screening and management guidelines specific for this disease have yet to be developed. The importance of understanding MAP, and thus diagnosing it and treating it appropriately, lies in its clinical similarity to other well‐characterized familial cancer syndromes. Its autosomal recessive nature directly contrasts the autosomal dominant nature of FAP; the diagnosis most commonly ruled out prior to its diagnostic consideration. This difference has implications regarding which family members to screen and when to screen them. Additionally, its clinical presentation is similar to, but distinct from, other familial cancer syndromes. Therefore, a distinct work‐up is likely necessary, however, one has yet to be independently established. Prospective studies on MAP have not yet been conducted. They would doubtlessly be invaluable to its accurate characterization and elucidation of its proper diagnosis and management.

## CONFLICT OF INTEREST

The authors declare no conflicts of interest.

## ETHICAL STATEMENT

This case report does not meet the requirements for institutional approval or patient consent and is exempt. Patient consent has been obtained and is available if requested.

## AUTHOR CONTRIBUTIONS

**Aaron Arroyave:** Conceptualization; data curation; investigation; writing‐original draft; writing‐review & editing. **Laurentia Nodit:** Conceptualization; data curation; investigation; project administration; visualization; writing‐original draft; writing‐review & editing. **Devin Clegg:** Conceptualization; formal analysis; investigation; project administration; visualization; writing‐original draft; writing‐review & editing. **Andrew Russ:** Conceptualization; data curation; formal analysis; investigation; project administration; supervision; visualization; writing‐original draft; writing‐review & editing.

## Data Availability

Data sharing is not applicable to this article as no new data were created or analyzed in this study.
